# A Randomized Trial of Nutrition and Exercise Treatment in Patients With Pancreatic and Non‐Small Cell Lung Cancer (NEXTAC‐TWO)

**DOI:** 10.1002/jcsm.13871

**Published:** 2025-06-16

**Authors:** Shuichi Mitsunaga, Tateaki Naito, Hisao Imai, Madoka Kimura, Satoru Miura, Hisashi Tanaka, Takuro Mizukami, Akira Imoto, Chihiro Kondoh, Hiroyuki Okuyama, Makoto Ueno, Shinsuke Shiotsu, Toshimi Inano, Haruka Chitose, Noriatsu Tatematsu, Taro Okayama, Takako Mouri, Miwa Sugiyama, Katsuhiro Omae, Takanori Kawabata, Keita Mori, Koichi Takayama

**Affiliations:** ^1^ Department of Hepatobiliary and Pancreatic Oncology National Cancer Center Hospital East Kashiwa Japan; ^2^ Division of Biomarker Discovery, Exploratory Oncology Research and Clinical Trial Center National Cancer Center Kashiwa Japan; ^3^ Cancer Supportive Care Center Shizuoka Cancer Center Shizuoka Japan; ^4^ Division of Respiratory Medicine Gunma Prefectural Cancer Center Gunma Japan; ^5^ Department of Respiratory Medicine, International Medical Center Saitama Medical University Saitama Japan; ^6^ Department of Thoracic Oncology Osaka International Cancer Institute Osaka Japan; ^7^ Department of Internal Medicine Niigata Cancer Center Hospital Niigata Japan; ^8^ Department of Respiratory Medicine Hirosaki University Hirosaki Japan; ^9^ Department of Clinical Oncology St. Marianna University School of Medicine Kawasaki Japan; ^10^ Department of Medical Oncology NTT Medical Center Tokyo Japan; ^11^ Second Department of Internal Medicine Osaka Medical and Pharmaceutical University Osaka Japan; ^12^ Department of Internal Medicine Aoyama Hospital Osaka Japan; ^13^ Department of Medical Oncology Toranomon Hospital Tokyo Japan; ^14^ Department of Medical Oncology Kagawa University Hospital Kagawa Japan; ^15^ Department of Gastroenterology Kanagawa Cancer Center Yokohama Japan; ^16^ Department of Respiratory Medicine Japanese red Cross Kyoto Daiichi Hospital Kyoto Japan; ^17^ Division of Nutrition Shizuoka Cancer Center Shizuoka Japan; ^18^ Nutrition Management Office National Cancer Center Hospital Tokyo Japan; ^19^ Department of Integrated Health Sciences Nagoya University Nagoya Aichi Japan; ^20^ Division of Rehabilitation Medicine Shizuoka Cancer Center Shizuoka Japan; ^21^ Graduate School of Nursing Kyoto Prefectural University of Medicine Kyoto Japan; ^22^ Division of Nursing Shizuoka Cancer Center Shizuoka Japan; ^23^ Department of Data Science National Cerebral and Cardiovascular Center Suita Japan; ^24^ Department of Biostatistics, Clinical Research Support Center Shizuoka Cancer Center Shizuoka Japan; ^25^ Department of Pulmonary Medicine Kyoto Prefectural University of Medicine Kyoto Japan

**Keywords:** cancer cachexia, elderly, low muscle mass, multimodal intervention, non‐small cell lung cancer, pancreatic cancer

## Abstract

**Background:**

In our previous study (NEXTAC‐ONE), the Nutrition and Exercise Treatment for Advanced Cancer (NEXTAC) program (including home‐based exercise and branched‐chain amino acid‐containing supplements combined with nutritional counselling) was shown to potentially prevent low muscle mass‐related disability in elderly cancer patients. This randomized controlled trial (NEXTAC‐TWO) was conducted to elucidate whether the NEXTAC program prolongs disability‐free survival in elderly patients with advanced pancreatic or non‐small cell lung cancer.

**Methods:**

This open‐label, multicentre, randomized phase II study was conducted at 15 Japanese hospitals. Patients aged ≥ 70 years, with pathologically proven advanced pancreatic or non‐small cell lung cancer, who were scheduled to undergo systemic chemotherapy for treatment‐naïve tumours were randomly assigned (1:1) to undergo observation or receive the NEXTAC program for 12 weeks. Randomization was performed by the minimization method, using performance status and types with cancer diagnosis and anticancer treatment as adjustment factors. The primary endpoint was disability‐free survival (period from randomization to the date patients were evaluated as needing care or death due to any cause). Key secondary endpoints were change in weight, muscle mass, physical activity, nutritional assessment, safety and survival. This trial was registered with the University Hospital Medical Information Network Clinical Trials Registry (UMIN000028801).

**Results:**

From 2017 to 2019, 131 patients were enrolled and randomly assigned to NEXTAC (*n* = 66) or control arms (*n* = 65, median age 76.0 years). After randomization, two patients in the NEXTAC arm declined further participation. As a result, 64 patients (median age 75.5 years) received at least one session of the NEXTAC program. The completion rate of the planned exercise and nutrition consultation sessions was 98.4% in the NEXTAC arm. Of the 129 patients, 91 (71%) had a disability (44 in the NEXTAC arm; 47 in the control arm). In the primary analysis, median disability‐free survival periods were 478 days (95% confidence interval [CI], 358–576) in the NEXTAC arm and 499 days in the control arm (95% CI, 363–604), with no significant differences between them (*p* = 0.884). The hazard ratio for disability‐free survival in the NEXTAC arm compared with the control arm was 0.970 (95% CI 0.642–1.465). There were no differences in the secondary endpoints between the two arms.

**Conclusions:**

The patients had good compliance with the 12‐week NEXTAC program but failed to show significant improvements in disability‐free survival as compared to observation alone. Further study on the progression of low muscle mass in the NEXTAC arm is needed.

## 1 Introduction

The number of elderly patients with pancreatic cancer (PC) and lung cancer is increasing worldwide. In Japan, in 2019, the number of new PC and lung cancer cases was 43 865 and 126 548 patients, respectively, with a high proportion of patients aged ≥ 70 years, accounting for 71.40% of patients with PC and 71.38% of those with lung cancer [1]. Low skeletal muscle mass commonly occurs in persons of advanced age and those with PC and non‐small cell lung cancer (NSCLC) and is a major cause of disability in these patients [2]. It is reportedly seen in 13.4% of men and 14.9% of women among the elderly Japanese population aged 65 years or older [2], 22.7% of men and 19.0% of women in the Korean population with advanced PC [3] and 52.6% of men and 90.9% of women in the Japanese population with advanced NSCLC [4]. A correlation between disability and low muscle mass has been found not only in elderly populations [2] but also in cancer patients [5]. Hence, since a large number of elderly patients with PC or NSCLC are expected to suffer low muscle mass‐related disability, identification of interventions to prevent disability is an important public health issue [6]. In a previous randomized controlled trial including an elderly population, preserving mobility by home‐based exercise reduced the risk of major disability as compared to a health education program [7]. Regrettably, however, no conclusive trial has as yet identified interventions to delay the onset of disability in advanced PC or NSCLC patients.

Cancer cachexia, which is one of the causes of low muscle mass, leads to progressive functional impairment [8] and impacts physical well‐being and quality of life (QOL), the ability to tolerate cancer therapies and gastrointestinal toxicity from systemic chemotherapy [9, 10, 11]. Fearon et al. proposed the definition of cancer cachexia as weight loss > 5% over the past 6 months, weight loss of > 2% and body mass index (BMI) < 20 kg/m^2^ or weight loss of > 2% and diagnosis of sarcopenia [8]. Its pathophysiology is characterized by a hypercatabolic state driven by reduced food intake and abnormal metabolism [8]. The estimated prevalence of cancer cachexia is 45.6% in PC patients and 37.2% in lung cancer patients [9]. Elderly patients with PC or NSCLC, therefore, face a risk of muscle wasting from not only old age, but also due to cachexia. In a previous study, muscle mass increased in cancer cachexia patients treated with an intervention consisting of home‐based exercise, an eicosapentaenoic acid‐based supplement combined with nutritional counselling and celecoxib as a nonsteroidal anti‐inflammatory drug, but with no improvement in mobility, which was attributed to poor compliance with the multimodal intervention [12]. We previously reported that home‐based exercise and branched‐chain amino acid (BCAA)‐containing supplements combined with nutritional counselling, named as the Nutrition and Exercise Treatment for Advanced Cancer (NEXTAC) program, were feasible in elderly patients with advanced PC or NSCLC (NEXTAC‐ONE) [13]. In that study, cachexia and low muscle mass were seen in 40% and 70% of the study population, respectively. About 90% of patients completed the daily exercise program and supplement consumption during the treatment period. Mobility increased after receiving the 8‐week NEXTAC program. The results indicated that the NEXTAC program has the potential to prevent low muscle mass‐related disability. We, thus, conducted this randomized controlled trial to elucidate whether conduct of the NEXTAC program for 12 weeks would prolong disability‐free survival in elderly patients with advanced PC or NSCLC (NEXTAC‐TWO).

## 2 Methods

### 2.1 Study Design

The NEXTAC‐Two study was a multicentre, randomized phase II study with elderly PC or NSCLC patients allocated to the intervention arm or control arm that was conducted at 15 centres in Japan between August 2017 and March 2021. Written informed consent was obtained from the patients before study enrollment. This study evaluated the effect of home‐based exercise and BCAA‐containing supplements combined with nutritional counselling (NEXTAC program), in comparison with no intervention. Details of the study design and rationale have been published previously [14]. During the screening period, after obtaining their written informed consent, patients were educated about wearing and handling an accelerometer (Lifecorder, Suzuken Co. Ltd., Nagoya, Japan). Patients who did not use the accelerometer for multiple days were judged as being ineligible and were not enrolled in the study. Patients who did not receive the first dose of first‐line chemotherapy within 14 days of enrollment were also excluded. Approval of the institutional review board at each participating institution was obtained before study initiation. This study was planned by the Cancer Cachexia Study Group of the Japanese Association of Supportive Care in Cancer, registered with the University Hospital Medical Information Network Center (UMIN000028801), and was supported by the Japan Agency for Medical Research and Development (AMED) under grant number JP18ck0106212. This study complied with the Declaration of Helsinki and the Japanese Ethical Guidelines for Medical and Health Research Involving Human Subjects.

### 2.2 Study Population

The eligibility criteria for study participation have been reported in our previous study [14]. The key eligibility criteria included age ≥ 70 years, Eastern Cooperative Oncology Group performance status (ECOG‐PS) 0, 1 or 2, availability of body weight data from 6 months earlier, pathologically proven PC or NSCLC, and scheduled to receive gemcitabine plus nab paclitaxel as first‐line chemotherapy for unresectable PC or systemic cancer therapy for treatment‐naïve advanced NSCLC (i.e., chemotherapy and/or targeted therapy and/or immunotherapy). The key exclusion criteria were symptomatic brain metastasis and bone metastasis with a high risk of fracture. Patients evaluated as needing care, based on the modified Katz index of Independence in Activities of Daily Living [15], were also excluded. The modified Katz index assesses the patient's independence in terms of four variables, including bathing, dressing, transferring and eating, defined as follows:

1. Bathing dependence: received help getting in or out of the tub or shower, received supervision with bathing, received help with washing more than one part of the body or was completely bathed by another person.
2. Dressing dependence: received supervision in dressing, received more assistance than having shoes tied or was completely dressed by another person.
3. Transferring dependence: received help or did not get out of bed.
4. Eating dependence: received supervision in eating, received more help than having bread buttered or meat cut or was completely fed by another person.’.

### 2.3 Randomization

Following screening and confirmation of eligibility, participants were enrolled and subsequently randomized to the NEXTAC arm or the observation arm. To achieve balance between the groups in terms of factors that could influence the outcomes, a minimization method was employed for the two‐arm randomization. The stratification factors used for adjustment were as follows: (1) ECOG‐PS (0–1, 2) and (2) type of cancer and treatment type/stage (lung cancer with chemotherapy, lung cancer with targeted therapy/immunotherapy, stage III PC [16] and stage IV PC). Since blinding was not possible due to the inherent nature of the interventions, both, participants and site investigators, were aware of the treatment allocations.

### 2.4 Twelve‐Week NEXTAC Program

Patients in the NEXTAC arm underwent NEXTAC program sessions at the initiation of systemic therapy (T1), 4 weeks later (T2), 8 weeks later (T3) and 12 weeks later (T4). Details about the exercise and physical and nutritional assessments have been published previously [14]. Briefly, physiotherapists assessed handgrip strength and the short physical performance battery (SPPB) score according to the study protocol at T1 and T4. The SPPB consists of three tests (balance test, 4‐m gait speed and the five times sit‐to‐stand test); a score equal to or less than 8 points indicates low physical performance [17]. Lumbar skeletal muscle mass was measured at T1 and T4 by analysing electronically stored computed tomography (CT) images using Slice‐O‐Matic software (version 5.0, Tomovision, Montreal, Quebec, Canada) [18]. Physical activity was assessed using an electronic pedometer and accelerometer (Kenz Lifecorder‐GS, Suzuken Co. Ltd., Nagoya, Aichi, Japan) [14]. The collected data included the number of daily steps, intensity of physical exercise during 4‐s intense workouts performed during the day, duration of device‐wearing and the daily duration of physical performance rated ≥ 1.8 or 3.9 metabolic equivalents (METs). Wearing the accelerometer for 5 h or more in a day was defined as a device‐wear day [13]. Nutritionists assessed the patients' nutritional status at every 4‐week visit and included measurement of body weight and the full version of the Mini Nutritional Assessment (full MNA) for estimating the ingested nutritional quantity [19] using a 24‐h recall method.

Patients in the NEXTAC arm received an exercise program session and a nutritional consultation session at each of the four time points mentioned above [14]. For each patient, a combined total of eight exercise and nutritional counselling sessions were conducted by physiotherapists and nutritionists, respectively. Lifestyle consultations to encourage the patients to engage in the activities daily were performed with medical doctors, physiotherapists or nurses. The exercise consisted of a muscle training program and a physical activity program. Muscle training programs included three to five of the following five exercises: sit‐to‐stand, calf raise, knee extension, knee raise and side‐leg‐raise with or without ankle weights. Patients performed each exercise at the optimized level three times a day. The physical activity program also included a lifestyle consultation to encourage the patients to engage in the activities daily. At every visit, the instructors educated the patients about the individually optimized muscle training programs and tried to motivate them to modify their lifestyle by increasing daily activities, according to the standardized manual. At every visit, nutritionists counselled the patients on how to improve their nutritional status and prescribed supplements rich in BCAAs (Inner Power, Otsuka Pharma Co. Ltd., Tokyo, Japan). Each pack of the prescribed supplement (139 kcal/125 g) contained branched‐chain amino acids (2500 mg), coenzyme Q10 (30 mg) and L‐carnitine (50 mg). Patients took one pack daily during the 12‐week NEXTAC program period.

Patients in the control arm underwent the same exercise, physical and nutritional assessments as patients in the NEXTAC arm. However, they did not participate in exercise program and nutritional consultation sessions and did not perform the prescribed exercises or take BCAA supplements.

Cachexia and low muscle mass were assessed at T1, based on our previous study [14]. Cachexia was defined as weight loss of > 5% during the previous 6 months or of > 2% in patients with a BMI < 20 kg/m^2^ or as the presence of low muscle mass. Low muscle mass was defined based on lumbar skeletal muscle index cutoffs of < 43.0 cm^2^/m^2^ for men with a BMI < 25.0 kg/m^2^, < 53.0 cm^2^/m^2^ for men with a BMI ≥ 25.0 kg/m^2^ and < 41.0 cm^2^/m^2^ for women.

### 2.5 Endpoints

The primary endpoint was disability‐free survival, which was defined as the period from group randomization to the date patients were evaluated as needing care, based on the modified Katz index. Information on Katz index items, including the care needed for bathing, feeding, dressing and moving from their bed to a chair, was obtained via in‐person or telephone interviews with the patients or caregivers every 8 weeks for 2 years after randomization. When the patient needed assistance for each of these four Katz variables, the patient was judged as having disability.

The NEXTAC‐Two trial evaluated physical and nutritional status as secondary endpoints. Physical assessments were conducted on the date of enrollment (T1) and 12 weeks later (T4). Further analyses included the secondary endpoints of QOL, assessed using the Japanese version of the EORTC QLQ‐C30 questionnaire ver.3.0 [20], overall survival (OS), that is, the period from group assignment to the date of death due to any cause, progression‐free survival (PFS), that is, the period from group randomization to the date of disease progression or death due to any cause, and treatment‐emergent adverse events (TEAEs), based on Common Terminology Criteria for Adverse Events v4.0 from the date of randomization to 12 weeks later.

### 2.6 Statistical Evaluations

This study was designed to confirm the superiority of NEXTAC over control observation in improving disability‐free survival. We assumed an expected disability‐free survival period of 12 months in the NEXTAC arm and 8 months in the control arm, based on the results of our previous study [4], in which disability‐free survival was 8 months in the study population of NSCLC cases at the Shizuoka Cancer Center. With a two‐sided alpha level of 0.2, 64 events in each arm were required to achieve a power of 80%. Hence, 130 patients were required with an enrolment period of 1.5 years and additional follow‐up period of 2 years.

Efficacy assessments were conducted in the full analysis set (FAS), which was defined as the population excluding those who were found to violate eligibility criteria after enrollment, those who never received the trial treatment and those with duplicate registrations. Assessments of safety endpoints were conducted in the safety analysis set (SAS), which included all participants who received at least one study assessment in the control arm or one study treatment in the NEXTAC arm.

Disability‐free survival, OS and PFS were estimated using the Kaplan–Meier method. The primary efficacy endpoint analysis was conducted using a log‐rank test to compare disability‐free survival between the control and NEXTAC arms. The confidence interval and significance level for the test were set at two‐sided 20%, in accordance with the rationale for the sample size calculation. A value of *p* < 0.05 was considered significant.

In the analysis of secondary endpoints, no multiplicity adjustments were performed, as planned. For the survival endpoints, the log‐rank test was used for group comparisons without confounding adjustments, and hazard ratios (HRs) were calculated using the Cox proportional hazards model. For other endpoints, Fisher's exact test or Student's *t*‐test was used for group comparisons. For the analysis of safety endpoints, the incidence and worst grade of adverse events were summarized by group.

## 3 Results

**FIGURE 1:**
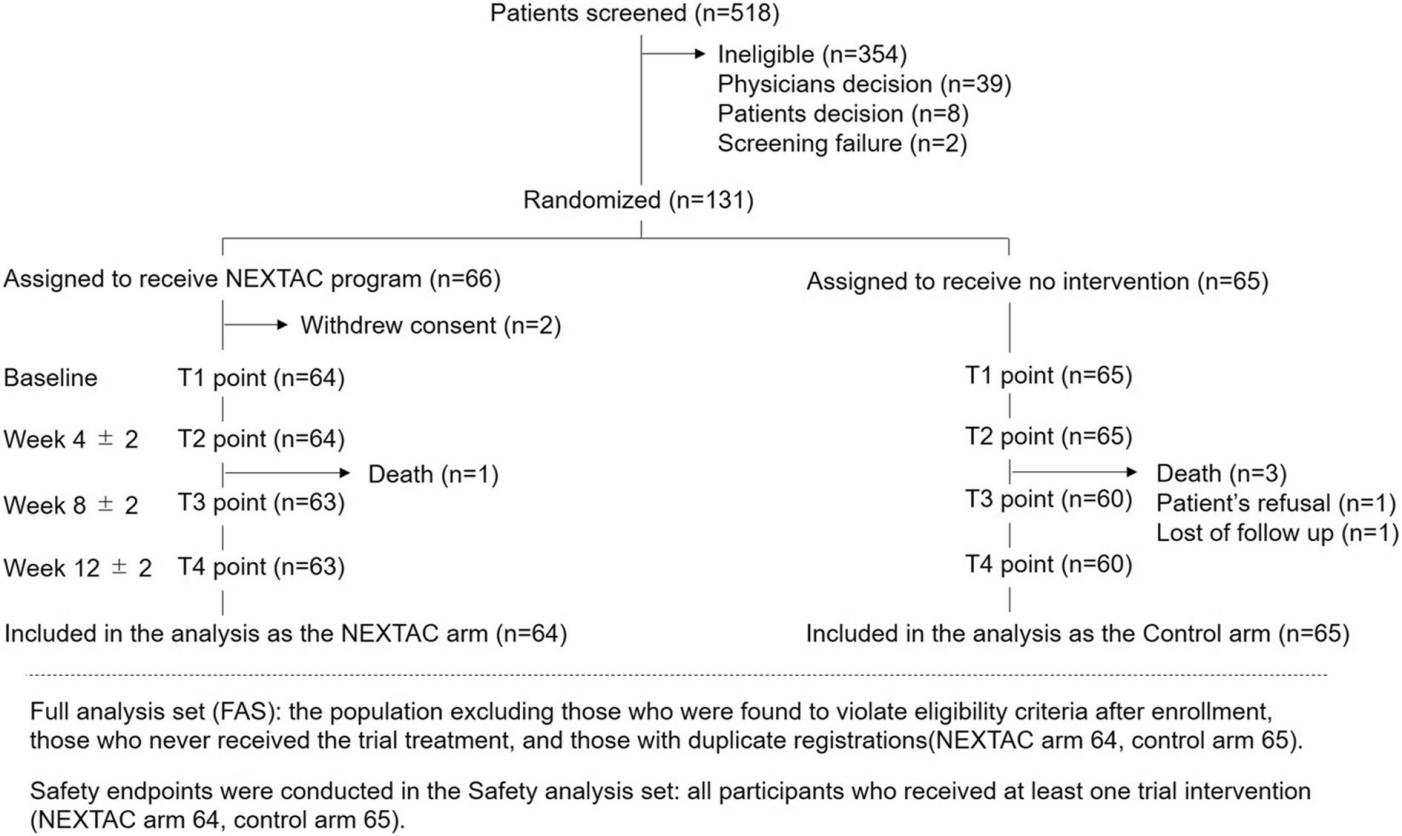
Patient inclusion in the study. The NEXTAC program consisted of exercise and branched‐chain amino acid‐containing supplements combined with nutritional consultation for a 12‐week period.

From November 2017 to March 2019, 131 patients were enrolled and randomly assigned to the NEXTAC arm (*n* = 66) or the control arm (*n* = 65) at 15 centres in Japan. After randomization, two patients in the NEXTAC arm declined further participation. As a result, 64 patients received at least one session of the NEXTAC program (Figure 1). The 129 evaluable patients included 38 PC and 91 NSCLC patients (Table 1). Cachexia [8] was identified in 67 (52%) of the 129 patients, and 90 (70%) patients had low muscle mass [21]. Cytotoxic chemotherapy had been administered to 80 (62%) patients, and targeted therapy or immunotherapy to 49 (38%) patients (detailed in Supplementary Table S1). The two arms were well matched with respect to baseline age, ECOG‐PS, cancer type, cachexia and low muscle mass (Table 1), EORTC‐QLQ‐C30 scores (Table S2), laboratory data (Table S3) and nutritional and physical parameters (Table S4). Patients randomized to the control arm had more advanced cancers. Median interval between randomization and the initiation of systemic chemotherapy was 2 days (IQR 1.0–2.3 days) in the NEXTAC arm and 2 days (1.0–4.0 days) in the control arm.

**TABLE 1:** Demographic characteristics of the patients at randomization.

Characteristics		NEXTAC arm *n* = 64	Control arm *n* = 65
Age (year)	Median (IQR)	75.5 (73.0–78.5)	76.0 (74.0–80.0)
Male sex	*n* (%)	45 (70.3)	41 (63.1)
ECOG‐PS	*n* (%)		
	0	29 (45.3)	25 (38.5)
	1	31 (48.4)	37 (56.9)
	2	4 (6.3)	3 (4.6)
Diagnosis	*n* (%)		
	Pancreatic cancer	19 (29.7)	19 (19.2)
	Non‐small cell lung cancer	45 (70.3)	46 (70.8)
Tumour status	*n* (%)		
	Stage III	12 (18.8)	6 (9.2)
	Stage IV	40 (62.5)	46 (70.8)
	Postoperative recurrence	10 (15.6)	12 (18.5)
	Relapse after radiation therapy	2 (3.1)	1 (1.5)
Cachexia	*n* (%)	31 (48.4)	36 (55.4)
Low muscle mass	*n* (%)	40 (65.6)	50 (78.1)
Systemic therapy	*n* (%)		
	Cytotoxic chemotherapy	40 (61.5)	40 (61.5)
	Targeted therapy or immunotherapy	24 (38.5)	25 (38.5)

*Note:* Tumour stage was evaluated using the TNM classification of malignant tumours, 8th edition. Cachexia was defined as weight loss of > 5% during the previous 6 months or of > 2% in patients with a body mass index (BMI) < 20 kg/m^2^ or the presence of muscle depletion. Sarcopenia was defined based on lumbar skeletal muscle index cutoffs of < 43.0 cm^2^/m^2^ for men with a BMI < 25.0 kg/m^2^, < 53.0 cm^2^/m^2^ for men with a BMI ≥ 25.0 kg/m^2^ and < 41.0 cm^2^/m^2^ for women.

Abbreviations: ECOG, Eastern Cooperative Oncology Group performance status; IQR, interquartile range.

**FIGURE 2:**
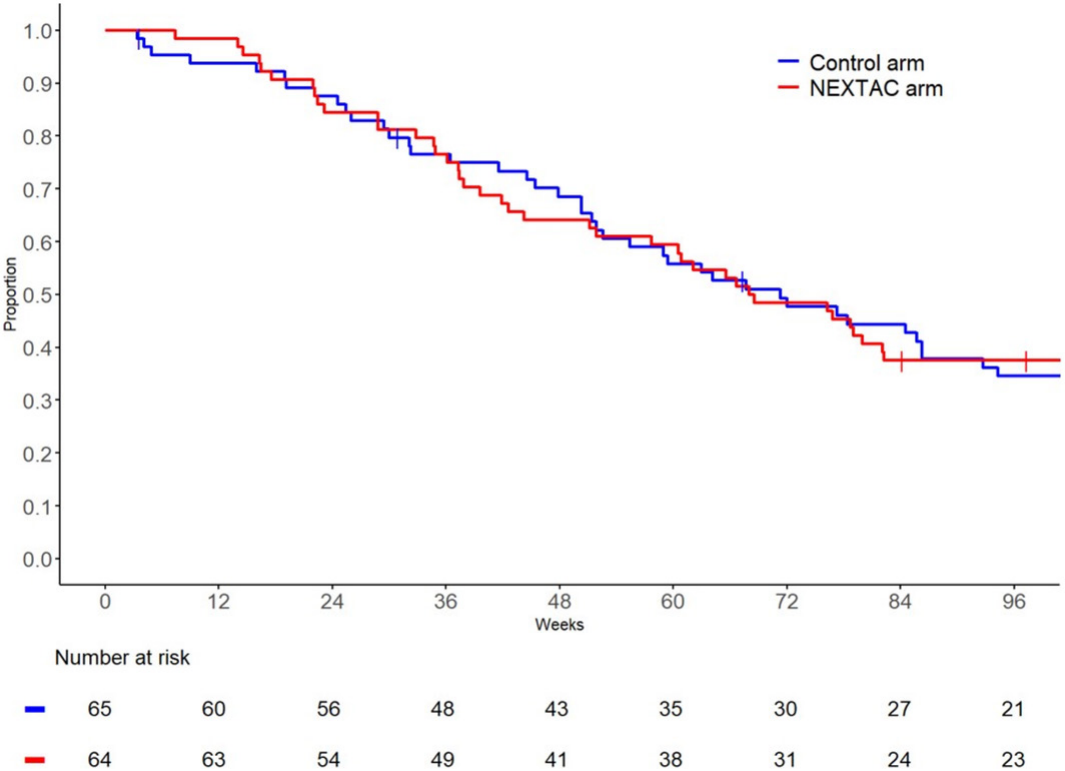
Kaplan–Meier estimates of disability‐free survival in the 129 evaluable patients according to treatment arm. The vertical marks along the curves indicate censored data.

The date for data cutoff were scheduled as March 31, 2021. Median duration of follow‐up for all evaluable patients was 18.2 months (IQR 10.1–28.3 months). Of the 129 patients, 91 (71%) were considered as having disability (44 in the NEXTAC arm, 47 in the control arm). The median disability‐free survival periods were 478 days (95% confidence interval [CI]: 358–576) in the NEXTAC arm and 499 days in the control arm (95% CI: 363–604), with no statistically significant difference between the two groups (*p* = 0.8841) (Figure 2).

**FIGURE 3:**
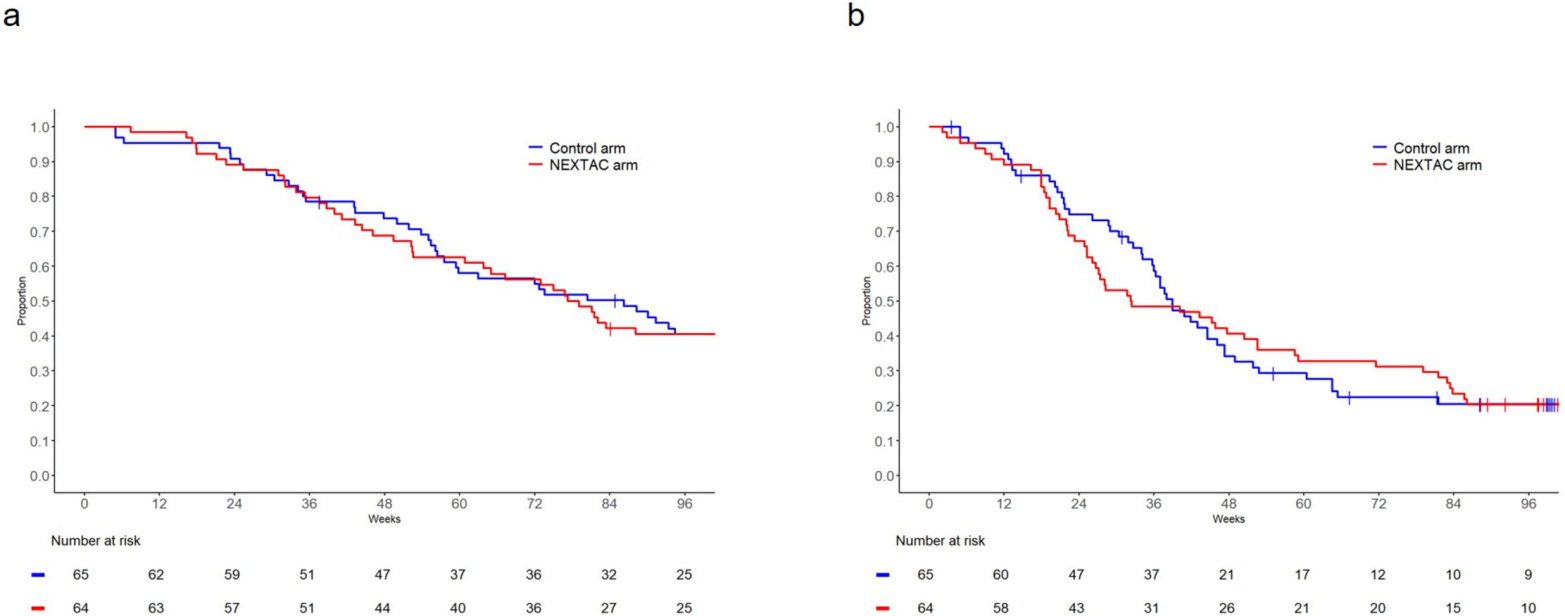
Kaplan–Meier estimates of overall survival (A) and progression‐free survival (B) in the 129 evaluable patients according to treatment arm. The vertical marks along the curves indicate censored data.

Changes in physical, nutritional and QOL measures during the 12 weeks were compared between NEXTAC and control arms (Table 2). Calorie intake increased in the NEXTAC arm as 127 kcal/day and decreased in the control arm as −41.6 kcal/day. Skeletal muscle indexes in the NEXTAC and control arms were decreased after the 12‐week NEXTAC program, as −1.777 and −0.686 cm^2^/m^2^, respectively. No differences were, however, observed in the secondary endpoints between T1 and T4 (Table S5). Median durations of OS and PFS were 547 days (95% CI: 368–802) and 226 days (95% CI: 177–368) in the NEXTAC arm and 604 days (95% CI: 395–767) and 273 days (95% CI: 239–323) in the control arm, with no statistically significant differences in OS and PFS between the two study arms (Figure 3).

**TABLE 2:** Changes in outcome measures.

Parameters	NEXTAC arm		Control arm		*p*‐value
	*n*	Change from T1 to T4	*n*	Change from T1 to T4	
Body weight (kg)	63	−1.23 ± 0.367	61	−0.736 ± 0.363	0.337
Skeletal muscle index (cm^2^/m^2^)	60	−1.78 ± 0.411	59	−0.686 ± 0.392	0.057
Full MNA score (point)	63	0.691 ± 0.610	60	0.408 ± 0.509	0.723
Calorie intake (kcal/day)	63	127 ± 59.0	60	−41.6 ± 50.9	0.032
Protein intake (g/day)	63	2.49 ± 2.50	60	−0.667 ± 2.45	0.369
Right hand grip strength (kg)	63	−1.38 ± 2.91	60	−1.01 ± 2.83	0.483
SPPB score (points)	62	−0.0806 ± 1.54	59	−0.170 ± 1.57	0.754
Five‐time sit‐to‐stand test (s)	61	0.189 ± 2.33	58	0.215 ± 2.66	0.954
Global health status/QoL (points)	63	0.529 ± 3.43	59	1.13 ± 3.75	0.906

*Note:* *p*‐values were calculated using the unpaired *t*‐test. A value of *p* < 0.05 was considered significant. Data are presented as the mean ± standard error.

Abbreviations: MNA, mini nutritional assessment; SPPB, short physical performance battery; QOL, quality of life.

The completion rates of the four exercise and four nutrition consultation sessions were 98.4% for both types of sessions in the NEXTAC arm (Table 3). The rates of performance of the full or modified exercise program and consumption of supplements in the 12 week intervention period were 85.6% and 88.0%, respectively. Values of the physical parameters measured using the accelerometer from T1 to T2 are shown in Table 3. Over the first 4 weeks, the proportions of days when the device was worn for 5 h or more were 100% in the NEXTAC arm and 94.6% in the control arm. Physical activities performed were significantly higher in the NEXTAC arm as compared to the control arm.

**TABLE 3:** Adherence to the nextac protocol from T1 to T4 and physical activity from T1 to T2.

Variables		NEXTAC arm	Control arm	*p*‐value
Number of the eight planned sessions attended	Median (IQR)	8 (8–8)	NA	
Patients who attended ≥ 6 of eight sessions	*n* (%)	63 (98.4)	NA	
Nutrition from T1 to T4				
Diet diary fill‐in days (%)	Median (IQR)	100 (86.3–100)	NA	
Supplement consumption days (%)	Median (IQR)	88 (73.0–97.2)	NA	
Adequate caloric intake^a^	*n* (%)	55 (87.3)	52 (85.3)	
Adequate protein intake^a^	*n* (%)	47 (74.6)	52 (85.3)	
Daily resistance training from T1 to T4				
Exercise diary fill‐in days (%)	Median (IQR)	94 (73.8–99.5)	NA	
Performance days (%)				
Full program	Median (IQR)	58.3 (17.1–83.6)	NA	
Self‐modified program	Median (IQR)	13 (3.6–32.5)	NA	
Full or modified program	Median (IQR)	85.6 (52.7–96.2)	NA	
Physical activity parameters using accelerometer from T1 to T2				
Proportion of days when the device was worn for ≥ 5 h (%)	Median (IQR)	100 (90.3–100)	94.6 (77.8–97.2)	< 0.001
Change in mean daily steps (steps/day)	Median (IQR)	556.2 (−378.5–1340.4)	−265 (−1312.4–236.9)	< 0.001
Change in mean time spent performing exercise ≥ 1.8 METs (min/day)	Median (IQR)	5.5 (−2.6–12.6)	−2.6 (−12.0–3.0)	< 0.001
Change in mean time spent performing exercise ≥ 3.9 METs (min/day)	Median (IQR)	0.2 (−2.5–2.0)	−0.5 (−3.1–0.5)	0.144

*Note:* *p*‐values were calculated using the unpaired *t*‐test. A value of *p* < 0.05 was considered significant.

Abbreviations: IQR, interquartile range; METs, metabolic equivalents; NA, not assessable.

^a^ Number (%) of patients whose caloric or protein intake during each time point met their requirements as assessed by nutritionists (NEXTAC arm, *n* = 63; Control arm, *n* = 61).

**FIGURE 4:**
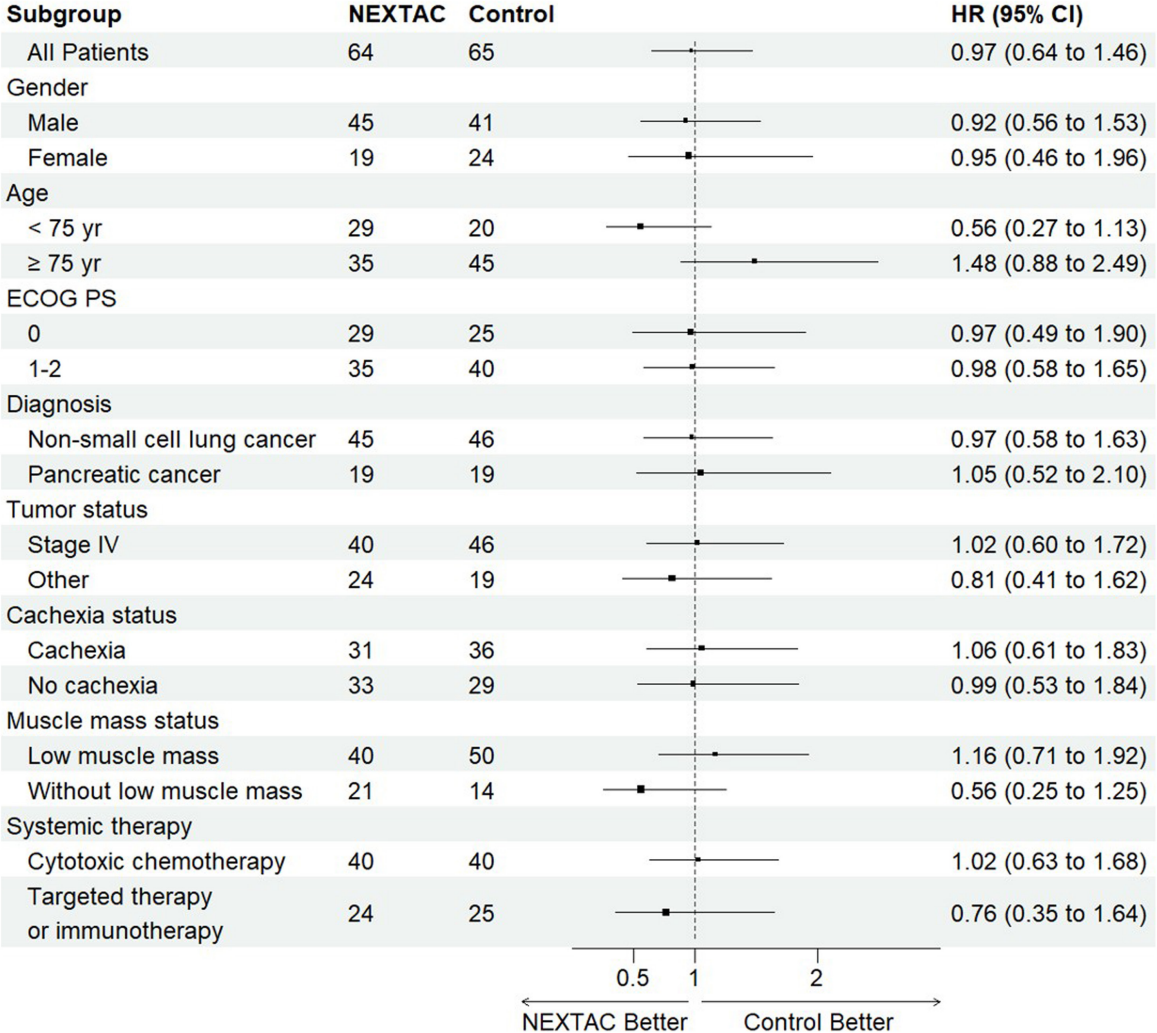
Forest plot of the treatment effect of disability‐free survival in subgroup analysis. The position of each square represents the point estimate of the treatment effect, and error bars represent 95% CIs. The sizes of the squares are proportional to the precision of the estimates. Tumour stage was evaluated using the TNM classification of malignant tumours, 8th edition. Cachexia was defined as weight loss of > 5% during the previous 6 months or of > 2% in patients with body mass index (BMI) < 20 kg/m^2^ or based on the presence of muscle depletion. Low muscle mass was defined based on lumbar skeletal muscle index cutoffs of < 43.0 cm^2^/m^2^ for men with a BMI < 25.0 kg/m^2^, < 53.0 cm^2^/m^2^ for men with a BMI ≥ 25.0 kg/m^2^ and < 41.0 cm^2^/m^2^ for women. ECOG‐PS, Eastern Cooperative Oncology Group performance status; HR, hazard ratio.

Among the 129 evaluable patients, the HR for disability‐free survival in the NEXTAC arm compared with the control arm was 0.970 (95% CI 0.642–1.465). Subgroup analyses of disability‐free survival among the FAS population showed no significant interactions between the NEXTAC arm and any of the variables (Figure 4). The benefits of NEXTAC on disability‐free survival, however, tended to be seen in patients younger than 75 years of age (HR 0.56, 95% CI 0.27–1.13) and in patients without low muscle mass (HR 0.56, 95% CI 0.25–1.25).

During the 12‐week period, four and two Grade 3 TEAEs were observed in the NEXTAC and control arms, respectively (Table 4). One case of Grade 1 fall was reported in the NEXTAC arm, and two cases were reported in the control arm, although no cases of Grade 2 or higher falls were observed. No events of fractures or cardiac‐related chest pain were reported as TEAEs.

**TABLE 4:** Treatment‐emergent adverse events.

	NEXTAC arm *n* = 64			Control arm *n* = 65		
	Grades 1–3	Grade 2	Grade 3	Grades 1–3	Grade 2	Grade 3
Fatigue	29 (45.3%)	3 (4.7%)	1 (1.6%)	21 (32.3%)	6 (9.2%)	0
Fall	1 (1.6%)	0	0	2 (3.1%)	0	0
Myalgia	5 (7.8%)	0	0	0	0	0
Arthralgia	7 (10.9%)	0	0	3 (4.6%)	0	0
Bloating	3 (4.7%)	0	1 (1.6%)	2 (3.1%)	0	0
Dyspnea	12 (18.8%)	1 (1.6%)	0	11 (16.9%)	1 (1.5%)	1 (1.5%)
Fracture	0	0	0	1 (1.5%)	0	1 (1.5%)
Pain	11 (17.2%)	3 (4.7%)	2 (3.1%)	9 (13.8%)	2 (3.1%)	0
Chest pain	0	0	0	0	0	0
Palpitations	29 (45.3%)	3 (4.7%)	0	0	6 (9.2%)	0

*Note:* Data are presented as the number (%). Treatment‐emergent adverse events (TEAEs) were evaluated according to the Common Terminology Criteria for Adverse Events, version 4.0. No Grade 4 TEAEs were observed in this study.

## 4 Discussion

In this randomized trial including elderly patients with advanced PC or NSCLC, the NEXTAC program demonstrated no benefit on disability‐free survival as compared to control observation. Median expected and actual disability‐free survival periods were 12 and 15.9 months, respectively, in the NEXTAC arm and 8 and 16.6 months, respectively, in the control arm. It is likely that the discrepancy between the expected and actual disability‐free survivals in both arms was a reason for the lack of intergroup difference in disability‐free survival. At the time of the reference study, only epidermal growth factor receptor (EGFR) gene testing was mandatory as driver gene testing prior to enrollment, and only 20% of patients had EGFR gene mutations and received first‐generation EGFR tyrosine kinase inhibitor (TKI) therapy. In contrast, during the NEXTAC‐TWO study period, more comprehensive gene profile testing became common, and 32 patients (24.4%) received molecularly targeted therapy for EGFR gene mutations, anaplastic lymphoma kinase fusion genes and ROS1 fusion genes. These treatments included second‐ and third‐generation EGFR TKIs ± angiogenesis inhibitors that were approved and available during the NEXTAC‐TWO study period. In addition, immune checkpoint inhibitors were approved for use in eligible patients during the study period and 18 patients (13.7%) received them as first‐line treatment, while many other participants received immune checkpoint inhibitors during later lines of chemotherapy. These advances in treating this patient population might have significantly improved the disability‐free survival rates in comparison with those predicted in the original protocol.

In this study, the 12‐week multimodal intervention, consisting of physical exercise training and nutritional counselling combined with BCAA supplements, was unsuccessful in increasing skeletal muscle mass. In a previous study on 12‐week exercise and nutritional treatment, gain in lean tissue mass was observed at the end of the 3‐month interventional period [20]. In another randomized controlled trial evaluating multimodal intervention consisting of nonsteroidal anti‐inflammatory drugs, eicosapentaenoic acid, exercise and nutritional counselling, along with oral nutritional supplements in cachexia patients with advanced PC or NSCLC, Solheim et al. [12] reported that loss of skeletal muscle mass was found in both multimodal intervention and control arms, although progression of low muscle mass in the intervention period was alleviated in the treatment arm. However, aggravation of skeletal muscle mass loss in the multimodal intervention arm as compared to the control arm was only observed in this study. We found advanced age to be a risk factor for low muscle mass in this study. The median ages of our study population were 75.5 years in the NEXTAC arm and 76.0 years in the control arm, which were high as compared to the reports on exercise and nutritional treatment by Solheim et al. (median age, 59.0 to 63.0 years) [12] and Storck et al. (mean age, 62.0 to 64.2 years) [20]. Indeed, in patients younger than 75 years, NEXTAC tended to exert beneficial effects on disability‐free survival as compared to no intervention (Figure 4). This suggests that the effects of multimodal intervention on low muscle mass might be limited in very elderly patients with advanced NSCLC or PC.

The duration of treatment efficacy might also have led to absence of an intergroup difference in disability‐free survival between the two study arms in our study. Storck et al. reported that gain in lean tissue mass was observed at the end of exercise for 3 months and disappeared 6 months after the start of the intervention in patients with advanced solid tumours [20]. In this study, the duration from the end of the intervention period (3 months) to the occurrence of disability (medians of 15.9 and 16.6 months, respectively, in the NEXTAC and control arms) was estimated as > 12 months, which might have led to coincidental overlapping of the time of disappearance of the effect of the intervention with the time the patients were faced with the risk of developing disability. Additionally, tumour progression after the start of chemotherapy seems to be associated with the progression of low muscle mass [21, 22]. In this study, PFS curves (Figure 3B) indicated an increase in the number of PFS events from week 24 to week 36 in the NEXTAC arm as compared to the control arm, which possibly resulted in an increased risk of disability due to skeletal muscle loss in the NEXTAC arm. This speculation needs further study to reveal the correlation between skeletal muscle loss and tumour progression.

CT is considered the gold standard for noninvasive assessment of muscle mass, but cutoff points for low muscle mass are not yet well‐defined [23]. In patients with cancer, the third lumbar vertebra on CT imaging correlates significantly with whole‐body muscle mass. As a result, CT‐L3 imaging is used in oncology to detect low muscle mass. The European Working Group of Sarcopenia in Older People (EWGSOP) recommends the assessment of muscle mass using dual‐energy X‐ray absorptiometry (DXA). However, lean tissue mass on DXA, as an indicator of muscle mass, is affected by hydration status [24] and the volume of intestinal content [25]. Patients receiving chemotherapy for advanced NSCLC or pancreatic ductal adenocarcinoma (PDAC) often experience pleural effusion, ascites or constipation secondary to opioid or 5‐HT3 antagonist treatment, which might contribute to bias in the measurement of muscle mass by DXA. These concerns might be resolved by analysing the correlation between lean body mass on DXA and the status of body water or the volume of intestinal contents in cancer patients.

In our previous 8‐week NEXTAC program, home‐based exercise programs and BCAA‐based supplements combined with nutritional counselling were selected as the NEXTAC program for maximizing compliance [13]. The rates of performance of the full or modified exercise program and consumption of supplements during the interventional period were 91% and 99% in that study [13] and 85.6% and 88.0% in the current 12‐week NEXTAC program. This suggests that the high compliance with the NEXTAC program among elderly PC or NSCLC patients was reproducible in the 12‐week intervention period. In both studies, nutritional and lifestyle consultations were conducted at every visit to encourage the patients to engage in daily activities and adopt the nutritional behaviours recommended in this study. These results indicated that the consultations with nutritionists, physiotherapists and nurses or medical doctors were effective in bringing about a behavioural transformation in feeding and physical activities. Lower toxicity is another feature of the NEXTAC program. In this study, Grade 2 or 3 TEAEs were found in 14 patients in the NEXTAC arm (21.9%) and 17 patients in the control arm (26.2%). A previous study of elderly people receiving a long‐term moderate‐intensity physical activity program showed that 198 of 818 participants (24.2%) developed symptoms that required at least 1 week of activity restriction [26]. Evaluation in that previous study showed that the rates of AEs resulting in restricted activity were similar among the NEXTAC arm, control arm and the elderly population receiving the physical activity program. We, thus, considered that the 12‐week NEXTAC program is feasible.

High compliance and less toxicity with the 12‐week NEXTAC program and the results of other studies using anticachectic agents [27, 28] have encouraged us to conduct combination therapy of our NEXTAC program with anticachectic agents. We are currently conducting a prospective study to evaluate the benefit of the 12‐week NEXTAC program combined with an appetite stimulant, ghrelin receptor agonist, for cancer cachexia (the NEXTAC‐THREE study). Previously, ghrelin receptor agonist reportedly led to gain of skeletal muscle mass in cachexia patients with NSCLC [28] and gastrointestinal cancer, including PC [29]. We hypothesized that the combination of NEXTAC and a ghrelin receptor agonist might prevent disability by causing gain in skeletal muscle mass in cancer cachexia patients. We hope that our prospective study will confirm the benefit of combining the NEXTAC program with an appetite stimulant.

This study was limited by the heterogenic population with regard to diagnosis of PC or NSCLC and type of systemic therapy as cytotoxic or noncytotoxic therapy. However, since randomization resulted in similar distributions of these variables in the NEXTAC and control arms, we consider that the effects of heterogenicity of the population on the comparative analysis between the two arms were limited.

In conclusion, despite the high compliance with the multimodal therapy among elderly patients with PC or NSCLC, the 12‐week NEXTAC program failed to produce significant improvements in disability‐free survival or gain in skeletal muscle mass as compared to control patients. The results also suggested that the effects of multimodal intervention on low muscle mass might be limited in very elderly patients with advanced NSCLC or PC. Further study on the progression of low muscle mass in the NEXTAC arm is needed.

## Ethics Statement

This study was approved by the ethics review committee of the Shizuoka Cancer Center (approval no. 29‐12‐29‐1‐5) and the institutional review boards of collaborating institutions and complied with the Declaration of Helsinki and the Japanese Ethical Guidelines for Medical and Health Research Involving Human Subjects. All the study subjects gave consent for study participation.

## Conflicts of Interest

S. Mitsunaga reports grants from Ajinomoto, Ono Pharmaceutical and Pfizer Inc. outside the submitted work; lecture fees from Ono Pharmaceutical, Otsuka Pharmaceutical and Meiji Seika pharmaceutical outside the submitted work; and a patent for a biomarker using free plasma amino acids to predict sarcopenia outside the submitted work. T. Natio is an associated editor for *The Journal of Cachexia, Sarcopenia and Muscle* and reports a grant from Otsuka Pharmaceutical outside the submitted work; a lecture fee from Ono Pharmaceutical outside the submitted work; and advisory fees from Ono Pharmaceutical and Pfizer outside the submitted work. S. Miura reports a lecture fee from Ono Pharmaceutical outside the submitted work. H. Tanaka reports lecture fees from Ono Pharmaceutical, Bristol Myers Squibb, AstraZeneca, Chugai Pharmaceutical, Boehringer‐Ingelheim, Eli Lilly, MSD, Takeda Pharmaceutical and Pfizer outside the submitted work. T. Mizukami reports a lecture fee from Ono Pharmaceutical outside the submitted work. C. Kondoh reports a lecture fee from Ono Pharmaceutical outside the submitted work. H. Okuyama reports a lecture fee from Otsuka Pharmaceutical outside the submitted work. M. Ueno reports a grant and a lecture fee from Ono Pharmaceutical outside the submitted work. H. Chitose reports lecture fees from Otsuka Pharmaceutical, Sanofi and Viatris outside the submitted work. K. Mori reports lecture fees from Chugai Pharmaceutical, Eli Lilly and Ono Pharmaceutical outside the submitted work. K. Takayama reports a grant from The Japan Agency for Medical Research and Development (AMED) under grant number JP18ck0106212 during the submitted work; grants from AstraZeneca, Chugai Pharmaceutical and Eli Lilly outside the submitted work; an advisory fee from AstraZeneca outside the submitted work; and lecture fees from AstraZeneca, Chugai Pharmaceutical, Boehringer‐Ingelheim, Eli Lilly, MSD‐Merck, Ono Pharmaceutical, Novartis, and Daiichi‐Sankyo outside the submitted work. No potential conflicts of interest were reported by the other authors.

## Supporting information


**Table S1** Types of chemotherapy.
Table S2. Quality of life parameters, assessed by the Japanese version of the EORTC QLQ‐C30 questionnaire ver.3.0 at T1 and T4 in the two study arms.
Table S3. Laboratory data at baseline.
Table S4. Nutritional and physical parameters at baseline.
Table S5. Body compositions, calorie intakes and serum levels of albumin and C‐reactive protein at T1 and T4.

## Data Availability

Qualified researchers may contact Tateaki Naito to request disclosure of individual‐level patient data.
