# Supplementary material for: A Randomized Trial of Nutrition and Exercise Treatment in Patients With Pancreatic and Non‐Small Cell Lung Cancer (NEXTAC‐TWO)
Source: J Cachexia Sarcopenia Muscle. 2025 Jun 16;16(3):e13871. doi: 10.1002/jcsm.13871 (PMC12169190; doi:10.1002/jcsm.13871)
Supplement: Supplementary file 1 — Table S1 Types of chemotherapy. Table S2. Quality of life parameters, assessed by the Japanese version of the EORTC QLQ‐C30 questionnaire ver.3.0 at T1 and T4 in the two study arms. Table S3. Laboratory data at baseline. Table S4. Nutritional and physical parameters at baseline. Table S5. Body compositions, calorie intakes and serum levels of albumin and C‐reactive protein at T1 and T4. [file JCSM-16-e13871-s001.docx]

# Supporting information

**Randomized controlled study evaluating nutrition and exercise treatment in pancreatic and non-small cell lung cancers**

***The Journal of Cachexia, Sarcopenia and Muscle***

Shuichi Mitsunaga, Tateaki Naito, Hisao Imai, Madoka Kimura, Satoru Miura, Hisashi Tanaka, Takuro Mizukami, Akira Imoto, Chihiro Kondoh, Hiroyuki Okuyama, Makoto Ueno, Shinsuke Shiotsu, Toshimi Inano, Haruka Chitose, Noriatsu Tatematsu, Taro Okayama, Takako Mouri, Miwa Sugiyama, Katsuhiro Omae, Takanori Kawabata, Keita Mori, Koichi Takayama

***Corresponding author***

Tateaki Naito

Cancer Supportive Care Center, Shizuoka Cancer Center, 1007 Shimonagakubo, Nagaizumi-cho, Sunto-gun, Shizuoka 411-8777, Japan

E-mail: t.naito@scchr.jp

**Table of Contents**

| Supplementary Table S1 | Types of chemotherapy |
| --- | --- |
| Supplementary Table S2 | Quality of life parameters, assessed by the Japanese version of the EORTC QLQ-C30 questionnaire ver.3.0 at T1 and T4 in the two study arms. |
| Supplementary Table S3 | Laboratory data at baseline |
| Supplementary Table S4 | Nutritional and physical parameters at baseline |
| Supplementary Table S5. | Body compositions, calorie intakes, and serum levels of albumin and C-reactive protein at T1 and T4 |

## Supplementary Table S1. Types of chemotherapy

| NEXTAC arm | n (%) |
| --- | --- |
| Afatinib | 4 (6.25) |
| Carboplatin＋Etoposide | 1 (1.56) |
| Carboplatin+nab-Paclitaxel | 4 (6.25) |
| Carboplatin＋nab-Paclitaxel+Nintedanib | 1 (1.56) |
| Carboplatin＋nab-Paclitaxel+Pemetrexed | 1 (1.56) |
| Carboplatin＋Paclitaxel+Bevacizumab | 1 (1.56) |
| Carboplatin＋Pemetrexed | 2 (3.13) |
| Carboplatin＋Pemetrexed+Pembrolizmab | 1 (1.56) |
| Carboplatin＋Tegafur/Gimeracil/Oteracil | 1 (1.56) |
| Cisplatin+Pemetrexed | 3 (4.69) |
| Crizotinib | 1 (1.56) |
| Docetaxel | 5 (7.81) |
| Gefitinib | 6 (9.38) |
| Gemcitabine+nab-Paclitaxel | 19 (29.7) |
| nab-Paclitaxel | 1 (1.56) |
| Osimertinib | 4 (6.25) |
| Pembrolizumab | 8 (12.5) |
| Pembrolizumab+Carboplatin＋nab-Paclitaxel | 1 (1.56) |
| Control arm |  |
| Afatinib | 2 (3.08) |
| Carboplatin＋Etoposide | 2 (3.08) |
| Carboplatin＋nab-Paclitaxel | 5 (7.69) |
| Carboplatin＋Pemetrexed | 1 (1.54) |
| Cisplatin+Pemetrexed+Pembrolizumab | 1 (1.54) |
| Crizotinib | 1 (1.54) |
| Docetaxel | 5 (7.69) |
| Docetaxel+Ramucirumab | 1 (1.54) |
| Erlotinib | 1 (1.54) |
| Gefitinib | 4 (6.45) |
| Gefitinib+Ramucirumab | 1 (1.54) |
| Gemcitabine+nab-Paclitaxel | 19 (29.1) |
| Osimertinib | 8 (12.1) |
| Osimertinib+Bevacizumab | 1 (1.54) |
| Pembrolizumab | 8 (12.4) |
| Pemetrexed | 1 (1.53) |
| Vinorelbine | 4 (6.15) |

## Supplementary Table S2. Quality of life parameters, assessed by the Japanese version of the EORTC QLQ-C30 questionnaire ver.3.0 at T1 and T4 in the two study arms.

|  | NEXTAC arm | | Control arm | | P value |
| --- | --- | --- | --- | --- | --- |
| Parameters | n | Mean ± standard error | n | Mean ± standard error |  |
| Global health status / QoL at T1 | 65 | 55.1 ± 2.92 | 64 | 57.2 ± 2.94 | 0.624 |
| Functioning scales at T1 |  |  |  |  |  |
| Physical | 65 | 86.7 ± 1.76 | 65 | 86.8 ± 1.76 | 0.933 |
| Role | 64 | 83.1 ± 2.84 | 65 | 86.2 ± 2.82 | 0.442 |
| Cognitive | 65 | 77.5 ± 2.52 | 63 | 79.4 ± 2.56 | 0.592 |
| Emotional | 65 | 81.8 ± 2.40 | 64 | 76.8 ± 2.41 | 0.147 |
| Social | 65 | 84.1 ± 2.23 | 64 | 87.2 ± 2.25 | 0.323 |
| Symptom scales / items at T1 |  |  |  |  |  |
| Fatigue | 65 | 27.5 ± 2.77 | 64 | 28.6 ± 2.79 | 0.775 |
| Nausea/vomiting | 65 | 5.12 ± 1.20 | 65 | 2.82 ± 1.20 | 0.176 |
| Pain | 65 | 18.7 ± 3.02 | 64 | 16.4 ± 3.05 | 0.592 |
| Dyspnea | 65 | 14.9 ± 2.74 | 65 | 18.5 ± 2.74 | 0.356 |
| Sleep disturbance | 65 | 24.1 ± 3.31 | 65 | 26.1 ± 3.31 | 0.662 |
| Appetite loss | 65 | 25.1 ± 3.69 | 65 | 20.0 ± 3.69 | 0.327 |
| Constipation | 65 | 23.1 ± 3.46 | 65 | 20.0 ± 3.46 | 0.529 |
| Diarrhea | 65 | 5.12 ± 2.10 | 64 | 6.77 ± 2.12 | 0.583 |
| Financial impact | 65 | 12.3 ± 2.55 | 64 | 12.5 ± 2.56 | 0.958 |
|  |  |  |  |  |  |
| Global health status / QoL at T4 | 63 | 55.8 ± 2.82 | 60 | 59.4 ± 2.89 | 0.372 |
| Functioning scales at T4 |  |  |  |  |  |
| Physical | 63 | 82.5 ± 2.35 | 59 | 78.5 ± 2.43 | 0.237 |
| Role | 63 | 80.2 ± 3.06 | 60 | 83.3 ± 3.14 | 0.471 |
| Cognitive | 62 | 85.1 ± 2.24 | 59 | 86.4 ± 2.30 | 0.672 |
| Emotional | 62 | 78.5 ± 2.35 | 60 | 81.1 ± 2.39 | 0.436 |
| Social | 63 | 86.0 ± 2.43 | 60 | 86.9 ± 2.50 | 0.783 |
| Symptom scales / items at T4 |  |  |  |  |  |
| Fatigue | 63 | 33.9 ± 2.95 | 60 | 30.5 ± 3.02 | 0.434 |
| Nausea/vomiting | 63 | 4.23 ± 1.68 | 60 | 4.45 ± 1.72 | 0.930 |
| Pain | 63 | 17.2 ± 2.90 | 60 | 15.0 ± 2.98 | 0.599 |
| Dyspnea | 63 | 23.8 ± 3.29 | 60 | 22.2 ± 3.37 | 0.737 |
| Sleep disturbance | 63 | 21.7 ± 3.27 | 60 | 15.5 ± 3.35 | 0.192 |
| Appetite loss | 63 | 27.5 ± 3.58 | 60 | 22.8 ± 3.67 | 0.357 |
| Constipation | 63 | 28.0 ± 3.71 | 60 | 20.6 ± 3.81 | 0.162 |
| Diarrhea | 63 | 15.3 ± 2.83 | 60 | 11.1 ± 2.90 | 0.298 |
| Financial impact | 63 | 10.0 ± 2.41 | 60 | 10.0 ± 2.47 | 0.987 |

P values were calculated using the unpaired-t test.

## Supplementary Table S3. Laboratory data at baseline

|  | NEXTAC arm | |  | Control arm | |
| --- | --- | --- | --- | --- | --- |
|  | n | Median (IQR) |  | n | Median (IQR) |
| White blood cells (/mm^3^) | 64 | 5890 (5030-7425) |  | 65 | 6180 (5390-7500) |
| Neutrophil count (/mm^3^) | 64 | 4051 (3191-5345) |  | 65 | 4182 (3291-5177) |
| Hemoglobin (g/dL) | 64 | 12.9 (11.5-13.8) |  | 65 | 12.7 (11.3-13.9) |
| Platelets (×10^4^/mm^3^) | 64 | 22.2 (18.7-26.7) |  | 65 | 23.1 (20.1-28.3) |
| Pseudo-cholinesterase | 63 | 247 (221-309) |  | 65 | 244 (207-311) |
| Pre-albumin (mg/dL) | 62 | 20.0 (14.2-24.3) |  | 65 | 20.2 (14.3-24.2) |
| Albumin (g/dL) | 64 | 3.9 (3.5-4.1) |  | 65 | 3.9 (3.5-4.1) |
| Total bilirubin (mg/dL) | 64 | 0.6 (0.4-0.8) |  | 65 | 0.6 (0.5-0.8) |
| Aspartate aminotransferase | 64 | 21.5 (19-28) |  | 65 | 22 (19-28) |
| Alanine aminotransferase | 64 | 17 (12.5-23) |  | 65 | 16 (12-24) |
| C-reactive protein (mg/dL) | 64 | 0.38 (0.09-1.70) |  | 65 | 0.33 (0.08-0.99) |
| Alkaline phosphatase (IU/mL) | 63 | 269 (217-356) |  | 65 | 238 (205-328) |
| Blood urea nitrogen (mg/dL) | 63 | 15.6 (11-19) |  | 65 | 14.1 (11.9-16.8) |
| Creatinine (mg/dL) | 64 | 0.7 (0.6-0.9) |  | 65 | 0.7 (0.6-0.9) |
| Na (mEq/L) | 64 | 140 (138-141) |  | 65 | 139 (137-141) |
| K (mEq/L) | 64 | 4.2 (4.05-4.5) |  | 65 | 4.3 (4-4.5) |
| Ca (mEq/L) | 63 | 9.3 (8.9-9.7) |  | 64 | 9.1 (8.9-9.4) |
| Glucose (mg/dL) | 64 | 111.0 (96.5-142.5) |  | 64 | 110.5 (101.0-147.0) |
| Percutaneous oxygen saturation (%) | 64 | 97.0 (95.5-98) |  | 65 | 97.0 (96.0-98.0) |
| IQR, Interquartile range. | | | | | |

## Supplementary Table S4. Nutritional and physical parameters at baseline

|  | NEXTAC arm | |  | Control arm | |
| --- | --- | --- | --- | --- | --- |
|  | n | Median (IQR) |  | n | Median (IQR) |
| Body weight (kg) | 64 | 57.1 (49.1-65.1) |  | 65 | 53.9 (47.0-64.5) |
| Skeletal muscle index (cm^2^/m^2^) | 61 | 41.8 (36.0-47.4) |  | 64 | 40.7 (34.8-45.9) |
| Full MNA score (points) | 64 | 23.8 (21.0-26.0) |  | 65 | 23.5 (20.5-26.0) |
| Calorie intake (kcal/day) | 64 | 1550 (1200-1850) |  | 65 | 1530 (1300-1860) |
| Protein intake (g/day) | 64 | 62.0 (50.0-72.5) |  | 65 | 62.0 (56.0-73.0) |
| Right hand grip strength (kg) | 64 | 28.5 (21.0-34.0) |  | 64 | 28.5 (21.0-34.0) |
| SPPB score (point) | 64 | 11.5 (10.0-12.0) |  | 64 | 11.5 (10.0-12.0) |
| Five-time sit-to-stand test (s) | 64 | 10.4 (8.1-12.0) |  | 64 | 10.4 (8.1-12.0) |
| Global health and QOL score (points) | 64 | 50.0 (37.5-75.0) |  | 64 | 54.2 (37.5-75.0) |

IQR, Interquartile range, MNA, Mini Nutritional Assessment; SPPB, Short Physical Performance Battery; QOL, quality of life.

Supplementary Table S5. Body compositions, calorie intakes, and serum levels of albumin and C-reactive protein at T1 and T4

|  | Parameters | | Timing | Median (IQR) | P value |
| --- | --- | --- | --- | --- | --- |
| NEXTAC arm | | |  |  |  |
|  | Body mass index (kg/m^2^) | | T1 | 22.3 (19.7-24.2) | 0.390 |
|  |  | | T4 | 21.7 (19.6-23.6) |  |
|  | Skeletal muscle index (cm/m^2^) | | T1 | 41.8 (35.6-47.5) | 0.231 |
|  |  | | T4 | 39.4 (34.4-44.6) |  |
|  | Calorie intake (kcal/day) | | T1 | 1550 (1200-1875) | 0.064 |
|  |  | | T4 | 1700 (1500-1940) |  |
|  | Albumin (g/dL) | | T1 | 3.85 (3.43-4.10) | 0.753 |
|  |  | | T4 | 3.80 (3.40-4.10) |  |
|  | C-reactive protein (mg/dL) | | T1 | 0.38 (0.09-1.80) | 0.455 |
|  |  | | T4 | 0.25 (0.07-0.72) |  |
| Control Arm | | |  |  |  |
|  | Body mass index (kg/m^2^) | | T1 | 21.8 (20.2-24.7) | 0.566 |
|  |  | | T4 | 22.0 (19.5-24.0) |  |
|  | Skeletal muscle index (cm/m^2^) | | T1 | 40.7 (34.6-46.0) | 0.425 |
|  |  | | T4 | 40.2 (34.2-43.3) |  |
|  | Calorie intake (kcal/day) | | T1 | 1530 (1300-1880) | 0.359 |
|  |  | | T4 | 1519 (1275-1820) |  |
|  | Albumin (g/dL) | | T1 | 3.90 (3.50-4.10) | 0.381 |
|  |  | | T4 | 3.70 (3.45-4.05) |  |
|  | C-reactive protein (mg/dL) | | T1 | 0.33 (0.08-1.06) | 0.368 |
|  | |  | T4 | 0.38 (0.095-1.36) |  |

P values were calculated using the unpaired-t test.
